# Diagnostic Endoscopic Ultrasound (EUS) of the Luminal Gastrointestinal Tract

**DOI:** 10.3390/diagnostics14100996

**Published:** 2024-05-11

**Authors:** Giovanna Impellizzeri, Giulio Donato, Claudio De Angelis, Nico Pagano

**Affiliations:** Gastroenterology Unit, Department of Oncological and Specialty Medicine, Azienda Ospedaliero-Universitaria Maggiore della Carità, 28100 Novara, Italy; g.impellizzeri@maggioreosp.novara.it (G.I.); eusdeang@hotmail.com (C.D.A.)

**Keywords:** endoscopic ultrasound, diagnostic indications, GI tract

## Abstract

The purpose of this review is to focus on the diagnostic endoscopic ultrasound of the gastrointestinal tract. In the last decades, EUS has gained a central role in the staging of epithelial and sub-epithelial lesions of the gastrointestinal tract. With the evolution of imaging, the position of EUS in the diagnostic work-up and the staging flow-chart has continuously changed with two extreme positions: some gastroenterologists think that EUS is absolutely indispensable, and some think it is utterly useless. The truth is, as always, somewhere in between the two extremes. Analyzing the most up-to-date and strong evidence, we will try to give EUS the correct position in our daily practice.

## 1. Introduction

Endoscopic ultrasonography (EUS) is a precious diagnostic tool since 1980. The field of EUS has then developed rapidly to encompass a wide range of indications and expanded from diagnostic to therapeutic procedures. EUS diagnostic indications in the gastrointestinal (GI) tract are mainly related to the study of intramural lesions and masses beyond the GI wall. The high-resolution capacity and low penetration depth of EUS allow for highly detailed images of the GI wall and the immediate surroundings to a depth of 4–5 cm [1,2]. Nowadays, we have many adjuvant tools in this field, such as fine-needle aspiration (FNA) and fine-needle biopsy (FNB) needles, contrast agents, elastography, and forward-viewing endoscopes, which contribute to maximizing the diagnostic power of EUS. Artificial intelligence (AI) is also a promising tool. In this comprehensive review, we summarize EUS diagnostic indications in the upper and lower GI tract.

## 2. Diagnostic EUS Equipment

### 2.1. Endoscopes

EUS uses a flexible video endoscope to place an ultrasound transducer in the GI tract, adjacent to an area of interest. The range of ultrasound (US) frequencies varies from 5.5 MHz to 20 MHz. Higher frequencies produce the highest resolution images, but with a limited depth of penetration, while lower frequencies provide a lower imaging resolution but greater penetration depth. Acoustic coupling with the mucosal surface is achieved by either: (i) inflating a water-filled balloon attached to the instrument tip, or (ii) infusing a volume of water directly into the lumen [3]. Four instrument types are currently used in clinical practice: the radial scanning endoscope, the linear one, the forward-view (FV) one, and the catheter probe (miniprobe). 

Radial transducers are used exclusively for diagnostic purposes. They have individual piezoelectric elements around the transducer distal tip in a 360° radial array, producing a US image in a plane perpendicular to the echoendoscope long axis. Linear EUS scopes have an operative channel and allow the real-time tissue sampling of targeted lesions and produce a US image in a parallel plane to the scope long axis (usually with a sector width between 100° and 180°) [4,5]. The FV echoendoscope is equipped with a front endoscopic and EUS view, allowing the deployment of needles and other devices through the working channel in a straight direction, like a gastroscope. The frontal view makes EUS exploration possible in post-surgical patients, permitting us to reach the afferent limb in Roux-en-Y gastric bypass, for example, to study the common bile duct (CBD) and the pancreatic head. Larghi et al. have also demonstrated how the FV echoendoscope is safe and highly effective in performing the FNA/FNB of solid and cystic lesions in the GI tract and in subepithelial lesions (SELs) [6,7,8]. The catheter probe is a small-caliber catheter (2–3 mm), passed via a standard endoscope working channel. It has a mechanical rotating transducer at its tip. High-frequency probes (20 MHz) are used to obtain very fine details of mucosal and submucosal lesions. The probe caliber allows it to pass through stenoses, for example, in stenosing cancers [3,9].

### 2.2. Needles

In some clinical scenarios, such as suspected disease localizations which may change the therapeutic strategy, suspected plastic linitis, or subepithelial lesions, the EUS imaging characterization itself is not enough to make a diagnosis. In these cases, we need to perform tissue sampling. We have different needles on the market, with different tip morphologies, stylet materials, and body flexibility. The choice of the correct needle is usually related to the endosonographer preference, the availability of rapid on-site evaluation (ROSE), and the target tissue to sample. Two main types of needles are available, for cytology and histology, respectively, for FNA and FNB. FNB needles are essential in the case of subsequent molecular studies, as metastatic lymph nodes or GI stromal tumors (GISTs). The sampling needle sizes are 19, 20, 22, and 25 Gauge (G). The European Society of Gastrointestinal Endoscopy (ESGE) Guidelines recommends the use of a 25 G or 22 G needle in the case of solid-mass or lymph-node sampling, with an equal recommendation between FNA and FNB needles. However, when a tissue core is the primary aim, the Society suggests the employment of larger needle calibers such as 19 G FNA or FNB needles or 22 G FNB needles [10].

## 3. Ultrasound Tools

Different brands of US processors are available on the market, all with incorporated additional imaging software including color Doppler, tissue elastography, and contrast-enhanced (CE) EUS. Moreover, another emerging tool is AI.

### 3.1. Color Doppler

Color Doppler US, providing lesion vascularization, can help differentiate between malignant from benign lesions and can also guide the needle trajectory during biopsies to avoid bigger vessels, thus preventing bleedings. The detecting flow imaging (DFI) technology was recently developed as an innovative Doppler US technique. With its unique algorithm, it can detect the microvascular vessels with very slow flow rates, without contrast agents and motion artifacts, thus helping to define lesions [11].

### 3.2. Elastography

Ultrasound elastography reports a measurement of tissue stiffness, evaluating the EUS image change before and after the application of a determined pressure exerted by the probe. As non-pathological tissue is soft, it deforms more than malignant tissue, which results in it being stiffer. Two different techniques are available: the qualitative and the semi-quantitative elastography. The first one differentiates between lesions according to their elasticity score on a color map, lending itself to a subjective evaluation, which can be extremely operator-dependent. “Strain ratio” and “strain histogram” elastography represent the semi-quantitative and more objective techniques, providing a numerical ratio of “regions of interest” (ROIs), such as lesion ROI/normal tissue, or through the strain histogram, which gives a graphical representation of the pixel color distribution of the target lesion stiffness.

### 3.3. Contrast-Enhanced EUS

Contrast-enhanced EUS (CE-EUS) is achieved through the direct intravenous administration of a new second-generation contrast (SonoVue Bracco SpA., Milan, Italy or Sonazoid Daiichi-Sankyo, Tokyo, Japan) followed by a 10–20 mL flush of saline. This generates the flow of 0.1–0.4 mm-diameter microbubbles inside blood vessels, thus magnifying the lesion vascular behavior, helping to perform a differential diagnosis [4,12].

### 3.4. Artificial Intelligence

AI based on deep-learning technology has progressed tremendously in recent years and it has already been applied to assist EUS for pancreatic diseases [13,14,15,16] and GI SELs, in particular, to differentiate gastrointestinal stromal tumors (GIST) from other SELs [17,18,19]. In this setting, AI is a promising tool, but we need further evidence.

## 4. Sedation

Since the endoscopic ultrasound is uncomfortable and time-consuming, sedation is necessary in order to improve patient satisfaction and the procedure success rate. Moderate sedation using benzodiazepines and opioids, similarly to standard diagnostic procedures such as for an upper endoscopy or colonoscopy, is the most widely used [20]. In recent years, there has been a lot of interest in propofol-balanced sedation (propofol in association with benzodiazepines and opioids, or only with benzodiazepines), with excellent results in the setting of EUS with or without FNA [21,22]. The longer procedural times compared to basic diagnostic procedures, the greater technical difficulty, the caliber of the instrument, and, therefore, the greater patient stimulation during maneuvers are all elements that make the propofol-balanced sedation ideal in the EUS setting.

## 5. Upper GI EUS Diagnostic Indications

### 5.1. Esophagus

#### 5.1.1. Esophageal Cancer

Esophageal cancer is the eighth-most common cancer and sixth-most common cause of mortality globally [23]. The two predominant types of esophageal cancer are squamous cell carcinoma (SCC) and adenocarcinoma. Most cases in Western countries are represented by adenocarcinoma arising from Barrett’s esophagus (BE). SCC is now mainly seen in developing countries and it accounts for 87% of all esophageal cancers worldwide [24]. Esophageal adenocarcinoma and SCC represent two different diseases with a separate pathophysiology and prognoses. However, the staging for both malignancies is determined similarly, with a combination of EUS and cross-sectional imaging. EUS’ role begins after the cross-sectional imaging has excluded distant metastasis (predominantly to the liver, lung, and bone). With the advent of less invasive interventions, including endoscopic mucosal resection (EMR) or submucosal dissection (ESD) for superficial cancers, the accurate clinical staging of esophageal cancer becomes critical in selecting the appropriate treatment options [23].

Locoregional staging is obtained through the evaluation of the tumor relationship with the wall layers and the presence of any lymphadenopathy, and, for this purpose, EUS is the study of choice. Computed tomography (CT) and magnetic resonance imaging (MRI) lack the ability to differentiate between the esophageal wall layers. CT, in particular, has a relatively poor sensitivity for determining cT (approximately 67% vs. EUS 85% for cT1a and 86% for cT1b). CT is the most accurate imaging modality, however, for identifying the invasion of adjacent structures (cT4). [25,26].

**Figure 1:**
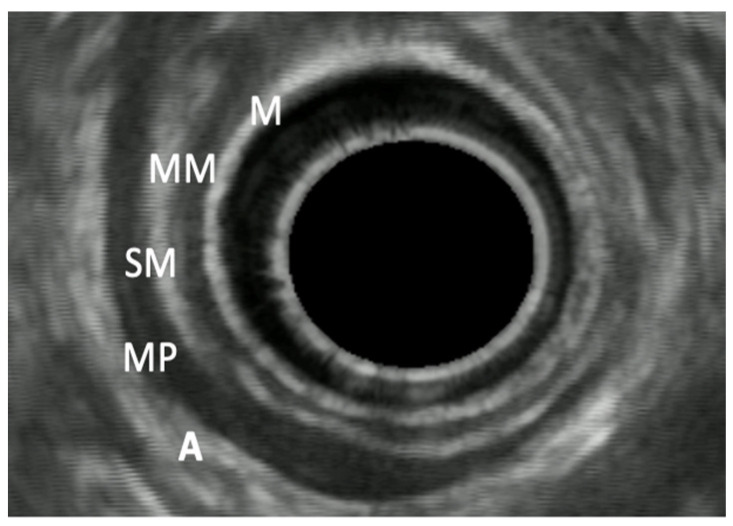
Endosonographic image revealing the five layers of the esophageal wall: mucosa (M), muscularis mucosa (MM), submucosa (SM), muscularis propria (MP), and adventitia (A).

EUS scanning resolves the GI wall into five layers (Figure 1): the mucosa (made by the interface between the transducer and the superficial mucosa and muscularis mucosae), submucosa, muscularis propria, subserosa, and serosa. The T staging of mucosal-based tumors depends on the depth of the invasion as follows: T1 lesions are superficial to the muscle layer, T2 lesions invade the muscle, and T3 lesions extend beyond the muscle, with T4 lesions invading adjacent organs or structures.

Superficial esophageal cancer is defined as a tumor invasion depth between Tis (high-grade dysplasia) to T1b (submucosal invasion). The lymph-node evaluation is another essential part of esophageal cancer staging. This is particularly true in T2 disease, where the presence of lymph nodes may shift treatment from surgical resection alone to include chemotherapy and radiation therapy [27]. EUS is the most accurate modality for the evaluation of locoregional lymphadenopathies. The endosonographic criteria suggestive of malignant lymph nodes include a hypoechoic sonographic pattern, sharply demarcated borders, round contours, and a width greater than 1 cm. Echogenicity is the most sensitive parameter for distinguishing benign from malignant nodes. However, only one-fourth of lymph nodes will have all four of these major features [28,29]. EUS can detect cervical, peri-esophageal, and peri-gastric lymphadenopathies. The overall accuracy of EUS for the nodal staging of esophageal cancer is 74% when used alone, and 90% if combined with FNA. When compared to CT, EUS-FNA has a better sensitivity and accuracy [30]. Lastly, EUS plays a role also in M-staging (distant lymph nodes, liver, peritoneum, and left adrenal gland metastasis). An important difference between the older classification (American Joint Committee on Cancer) system and the current, affecting the utility of EUS in differentiating M0 from M1 disease, is that the involvement of a celiac lymph node is now considered regional (N) disease and no longer M1 [31].

#### 5.1.2. EUS in Superficial Esophageal Cancer

Esophageal cancer confined to the epithelium (T1a) has a much more favorable prognosis than that extending into the submucosa, which contains lymphatic vessels promoting the spread of malignant cells to regional lymph nodes [32,33]. In one case series involving 3963 patients from the National Cancer Database (NCDB), the risk of lymph-node metastasis was 5.0% for T1a and 16.6% for T1b lesions [34,35]. Given the difference in prognosis and treatment options, it is crucial to make the distinction between superficial (T1) and advanced (T2 or greater) lesions. In superficial esophageal adenocarcinoma, endoscopic therapy can be curative in 94% of cases, with a 5-year survival rate of 98% [36]. The overall accuracy of EUS for staging esophageal cancer ranges between 73% to 93% depending on the stage, with the worst results in superficial ones [37]. A meta-analysis of 12 studies was conducted to evaluate whether EUS correctly predicts the T stage of esophageal high-grade dysplasia or intramucosal adenocarcinoma, compared to specimens obtained after endoscopic or surgical resection. EUS had a T stage concordance of only 65%, which led to the conclusion that it is not accurate for the staging of superficial esophageal cancer [38].

Factors associated with T-overstaging were the larger size of the lesion (>2 cm), poor differentiation, and protruding morphology, so caution should be taken when evaluating cancers with these features [39]. This fits for both esophageal cancer histotypes. A prospective study confirmed that the EUS addition is not associated with improvements in the diagnostic performance of non-magnifying endoscopy (ME) and ME for differentiating between shallowed and deeper submucosal cancers. Therefore, the results did not support the routine use of EUS to determine an indication for endoscopic resection after non-ME and ME evaluation in T1 SCC. The endoscopic resection of lesions not suspicious for submucosal invasion at standard high-definition endoscopy undeniably gives a more precise staging in addition to the therapeutic benefit. Therefore, the value of EUS in these cases seems to be limited. Accordingly, the ESGE guidelines limit the role of EUS prior to esophageal squamous lesion ESD to those lesions suspected at ME for deep mucosal-superficial submucosal invasion (m3-sm1). Similarly, EUS is reserved only in the suspicion of deep submucosal invasion (deep ulceration and markedly elevated borders) in BE-associated lesions [40]. In short, the endoscopic resection can be both diagnostic and therapeutic for early esophageal cancers, and EUS can be avoided.

#### 5.1.3. EUS in Advanced Esophageal Cancer

**Figure 2:**
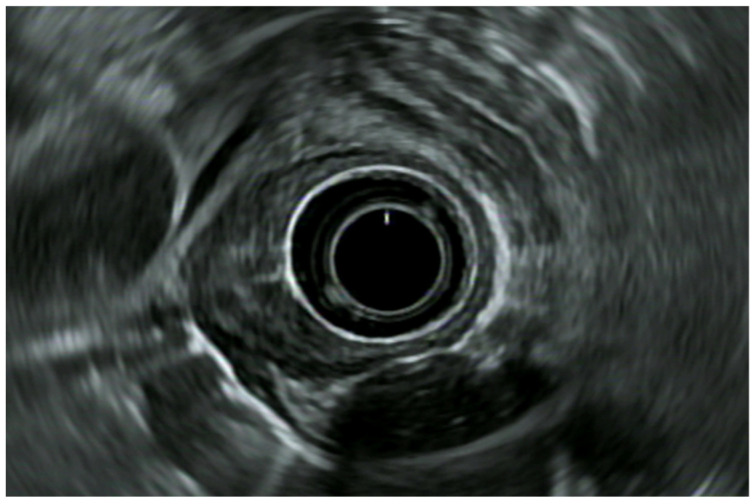
Radial EUS image of an esophageal lesion invading the adventitia (uT3).

EUS accuracy increases with a more advanced disease. It can indeed accurately stage 67% of T1 tumors, 33% of T2 lesions, and 100% of T3 ones (Figure 2) [41]. In M0 advanced esophageal cancer, the EUS locoregional staging is crucial in order to guide the therapeutic strategy (upfront surgery vs. radio-chemotherapy).

EUS in esophageal cancer staging has two limitations: the difficult assessment of stenotic lesions and the staging after neoadjuvant chemotherapy. In obstructive lesions (20–36% of cancers), the echoendoscope may not be able to pass the malignant stricture. This scenario precludes a proper analysis of the staging, providing a significantly reduced accuracy in the assessment of both the T and N stage (28% and 72%, respectively), compared to fully traversable lesions (81% and 86%, respectively) [42]. Options to improve diagnostic assessment include the use of dilators and miniprobes. However, the accuracy of high-frequency miniprobes declines in cancers with a deeper invasion depth [39]. As far as the staging after neoadjuvant therapies is concerned, a previous meta-analysis revealed a poor sensitivity and specificity for both the T and N staging in patients undergoing EUS after neoadjuvant chemotherapy. This is thought to be related to the local inflammation and fibrosis caused by chemotherapy or radiation. While chemoradiation treatment can decrease the tumor size, it is not accompanied by the restoration of the normal esophageal mucosa [43]. One multicenter study evaluating 138 patients before and after neoadjuvant therapy showed that EUS was able to adequately detect residual disease in 90% of patients 12 weeks following therapy. Specifically, EUS was also able to detect residual thickness and residual tumor areas in this setting, thus making EUS a reliable tool [44].

### 5.2. Esophageal Subepithelial Lesions

GI SELs include various non-neoplastic and neoplastic lesions which typically arise from a non-mucosal layer within the wall. Only 15% of these lesions are malignant at the time of diagnosis [45]. When small, SELs rarely cause signs or symptoms and are typically incidental findings. However, depending on their location and size, some may cause symptoms such as dysphagia, overt or occult GI bleeding, and chronic anemia. Because their location typically precludes making a diagnosis through simple mucosal biopsies, they often present a diagnostic challenge. Nonetheless, through a combination of typical radiographic, endoscopic, and endosonographic features, coupled with the judicious use of EUS-guided sampling, a definitive diagnosis can be made in most cases. The most common GI SELs are lipomas, followed by GI stromal tumors (GISTs) and leiomyomas. In the esophagus, the most common SELs are leiomyomas, granular cell tumors, duplication or bronchogenic cysts, varices, and metastases [46].

**Figure 3:**
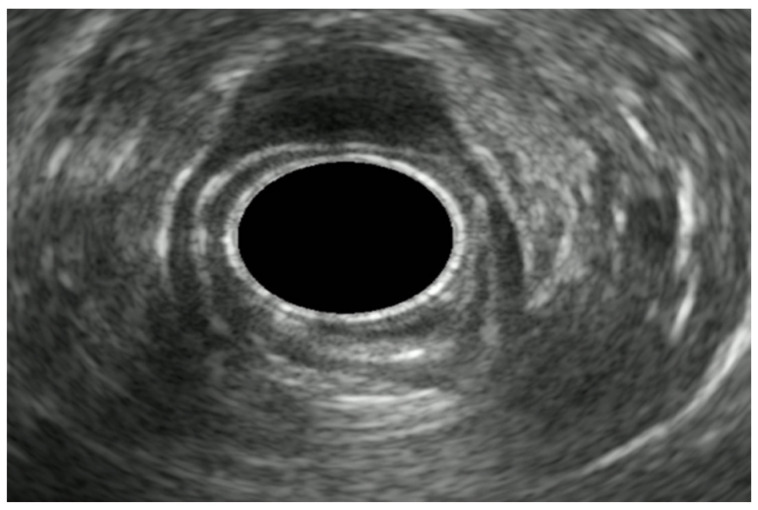
Esophageal leiomyoma arising from the muscularis propria.

Leiomyomas are benign tumors which originate from either the II or IV layer (Figure 3). Their EUS imaging features are similar to those of GISTs (hypoechoic), which are extremely rare in the esophagus (<5% of all GISTs). Leiomyomas are diagnosed based on their histology (desmin-positive, α-SMA-positive, C-kit-negative, and CD34-negative) [47,48].

Granular cell tumors (GCTs) are rare lesions of Schwann cell origin. Typical GCTs appear as a hard, isolated grey-white submucosal or mucosal nodule with normal overlying mucosa in the distal esophagus. At EUS, they appear as homogeneous or mildly heterogeneous, hypoechoic nodules growing within the mucosa or submucosa. Although their histological behavior is usually benign, 1–2% are malignant, and a larger size and growing rate can be malignancy predictors. They can occasionally grow during follow-up and show an invasion into the muscularis propria, or be associated with dysphagia, and, in these cases, an endoscopic resection can be considered [49,50].

Duplication or bronchogenic cysts can have any location in the GI wall and can also be extrinsic. They harbor an extremely rare malignant potential and their endosonographic appearance (anechoic lesion with sharp borders, and no Doppler signal in the submucosal layer or external) is pathognomonic for diagnosis [46].

Most esophageal SELs are, therefore, non-malignant.

## 6. Stomach

### 6.1. Gastric Epithelial Cancer

Gastric cancer is the fourth most commonly occurring cancer in the world, with a declining incidence in Western countries. It is the second most common cancer-related cause of death (10% of all cancer deaths) and almost 56% of the new cases derive from Eastern Asia [51].

Two-thirds of the incident cases and deaths occur in developing countries [52].

Radical surgery still represents the standard of care for curative intent [53], but, in the last decades, new approaches are gaining importance. Endoscopic treatment with ESD has grown as an alternative to surgery for early gastric cancer (EGC) in the presence of favorable characteristics of the lesion, like a good differentiation, superficial involvement, diameter less than 2 cm, and absence of ulceration [54,55,56,57]. In more advanced lesions, adjuvant and neoadjuvant chemotherapy regimens (combined or not with radiotherapy) have been shown to increase survival [58,59,60,61].

Tailoring the strategy in single cases requires accurate staging procedures in order to guarantee personalized medicine. As for all solid tumors, the disease stage for gastric cancer is defined by the TNM classification.

The T stage refers to the tumor invasion through the layers of the gastric wall with the T1 tumor invading only the mucosal or submucosal layer, T2 invading the muscularis propria, T3 the subserosa, and T4 serosa or adjacent organs. The N stage refers to the regional lymph-node involvement, with N0 if no nodes are involved, and N1 to 3 in the case of an increasing number of involved nodes. The M stage indicates the presence or absence of distant metastasis, such as hepatic or peritoneal metastasis (M0–1) [62]. Advanced cases (i.e., T3–T4 tumors or tumors with lymph-node metastasis) are treated with neoadjuvant (preoperative) chemotherapy (or radiotherapy, or both) [58,59,60]. On the other hand, early gastric cancer (T1 tumors) with no lymph-node involvement (N0) can be treated with an endoscopic rather than surgical resection [54,55,56,57]. Computed tomography (CT) is currently the most frequently used radiological tool for the preoperative staging [63,64]. The CT accuracy is high mainly for distant metastases, whereas it is much lower in the locoregional staging, ranging in most series from 65% to 85% [65,66,67,68,69]. In a meta-analysis, the CT scan showed a sensitivity and specificity of 77 and 78% for the identification of lymph-node status. Magnetic resonance imaging (MRI) or positron emission tomography (PET) do not seem to provide better results [70,71,72]. EUS provides instead a better accuracy in the locoregional staging [73,74,75].

The morphologic growth pattern of GC is defined according to the Borrmann classification as polypoid (lesion type I), fungating (lesion type II), ulcerated (lesion type III), or infiltrative (lesion type IV). The group of early lesions is further classified according to the Paris classification as protruded (type 0-I), slightly elevated (called type 0-IIa), flat (type 0-IIb), slightly depressed (type 0-IIc), and excavated (type 0-III), and combined types can be encountered. The Paris classification is related to the depth of the submucosal invasion [76,77].

The jistology of gastric cancer follows the Lauren classification which distinguishes the “intestinal type” from the “diffuse type”, characterized by pangastric infiltration (called “signet ring cells”) and a worse prognosis. If the tumor extends diffusely to the gastric wall through lymphatic invasion, it can produce gastric wall thickening without causing a mass effect (thus called “plastic linitis”) [78,79,80].

### 6.2. EUS in Early Gastric Cancer

**Figure 4:**
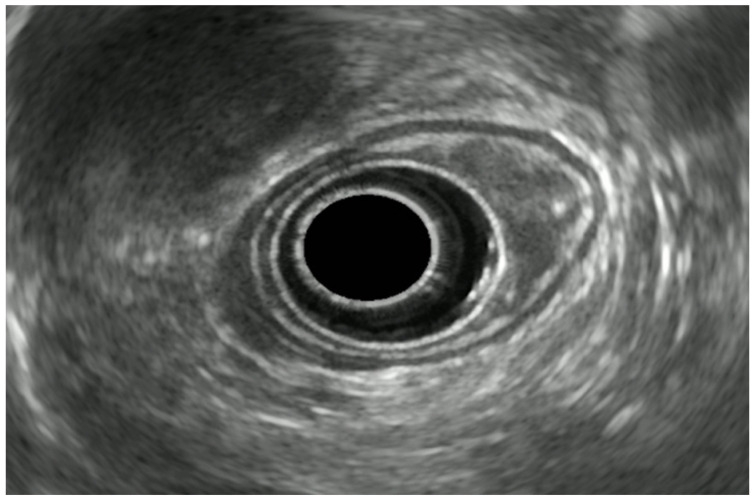
T1b gastric cancer (superficial submucosal invasion).

Many studies have been conducted to investigate the accuracy of EUS in evaluating the depth of the invasion in early lesions (Figure 4), but the results have a high grade of variability, and an accuracy rate between 64.8% and 92% has been computed. A recent meta-analysis of 17 articles and 4525 lesions showed a limited value for EUS in the evaluation of the invasion depth in EGC, with a sensitivity of 87% and specificity of 67%. Overall, the EUS over-staging rate in differentiating between T1a (mucosal or superficial submucosal invasion, SM1) vs. T1b (deep submucosal invasion SM2) was 32.8%, while the overall under-staging rate of T1b was 29.7%. The conclusion was that EUS has no better results than the high-definition endoscopic evaluation [81,82,83].

A radial EUS-scope is more frequently used for this indication, but, recently, the linear EUS-scope is more widespread because it allows us to perform biopsies. Lan et al. compared the accuracy of EUS with radial or linear scopes in EGC staging, concluding that the linear scope is more accurate in establishing SM involvement, with a diagnostic accuracy of 90.9% vs. 69.2%. The sensitivity was comparable (92.3% linear vs. 90.9% radial), while the specificity was higher in the linear group (90.0% vs. 60.7%). Therefore, the echoendoscope choice can be a risk factor of the incorrect T1b staging in EGC [84].

A Japanese study [85] highlighted the miniprobe accuracy of 72.3% in evaluating the mucosal or submucosal involvement in EGC. The main causes of mistakes were attributed to inflammation associated with ulcerated lesions, the presence of benign cystic glands in the submucosal layer, and the attenuation of the high-frequency ultrasound beam. A more recent German study [86] evaluated the diagnostic accuracy of miniprobes vs. linear EUS in the staging of tumors of the upper digestive tract. Miniprobes were superior to conventional EUS in the staging of early tumors, in particular, T1 tumors (accuracy of 81% vs. 56%), with a combined accuracy of both of 82%. A correct evaluation of the lymph-node involvement with miniprobes and the conventional EUS was obtained in 76% and 71% of cases, respectively, while the combined accuracy of the two methods was 73%.

### 6.3. EUS in Advanced Gastric Cancer

**Figure 5:**
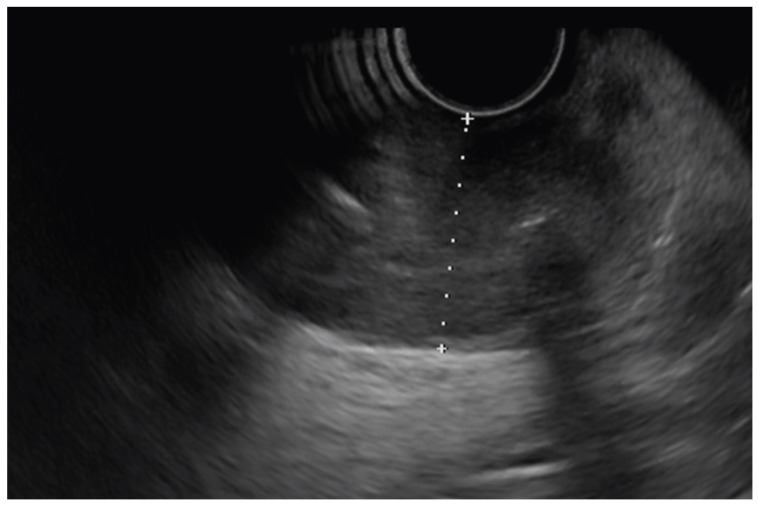
Advanced gastric cancer (linear scope view).

Surgery is still the only potentially curative strategy in locally advanced disease (Figure 5). Neoadjuvant treatment on locally advanced GC has shown a positive impact, improving survival and reducing recurrence according to the recent evidence [87,88,89].

Neoadjuvant treatment is indicated in lesions extending to or beyond the muscularis propria (T2) and/or in the presence of malignant EUS-visible lymph nodes (N+): stages IB–IIIC. EUS plays an important role in this setting, allowing us to overcome the limitation of CT scan. EUS showed a good accuracy (>90%) in various studies in establishing the T stage and N stage [90,91,92].

Costa et al. [93] found an accuracy of 86% in the selection of patients with gastric adenocarcinoma for neoadjuvant therapy (T2 and/or N+). The sensitivity and specificity were 88.5% and 83.1% with a better performance in the proximal location and intestinal type.

### 6.4. EUS in Gastric Linitis Plastica

Limited or diffuse (linitis plastica) infiltrating cancers (Bormann type IV) may comprise 30 to 50% of all gastric malignancies. In about one-third of such cases, there is no mucosal breakthrough by the cancer itself so that its endoscopic recognition may be difficult. A few patients with gastric linitis plastica remain undiagnosed by endoscopic biopsy and brush cytology, which only sample surface tissues [94]. There are some studies showing the rate of missed biopsies is as high as 30 to 55% [95,96]. In these cases, EUS can help the diagnosis, identifying a diffuse thickening of the involved gastric wall, which is almost pathognomonic, and through deeper layers sampling. The diagnostic yield of EUS-FNA in these cases is almost 60% [97,98], thus making EUS very useful in this setting.

### 6.5. Gastric Subepithelial Lesions

Gastric SELs are mostly incidental findings in asymptomatic patients, identified during upper endoscopies (0.3–2%) or cross-sectional imaging [99].

The first step in evaluating a gastric SEL is to differentiate between intra- or extramural lesions. Common structures that can cause an extrinsic compression are the xyphoid bone, the splenic artery (fundus), the left hepatic lobe, the spleen/accessory spleen (fundus, upper body), the gallbladder (antrum), or pathological abdominal masses (such as tumors, pancreatic pseudocysts, and enlarged lymph nodes), and vessel aneurysms [46,100,101]. EUS achieves a 92% sensitivity in recognizing extrinsic compressions. For this purpose, changing probe frequencies is helpful, in order to, first, clearly define the extramural space (lower frequencies), and then the integrity of the gastric wall (higher ones) [102,103].

**Figure 6:**
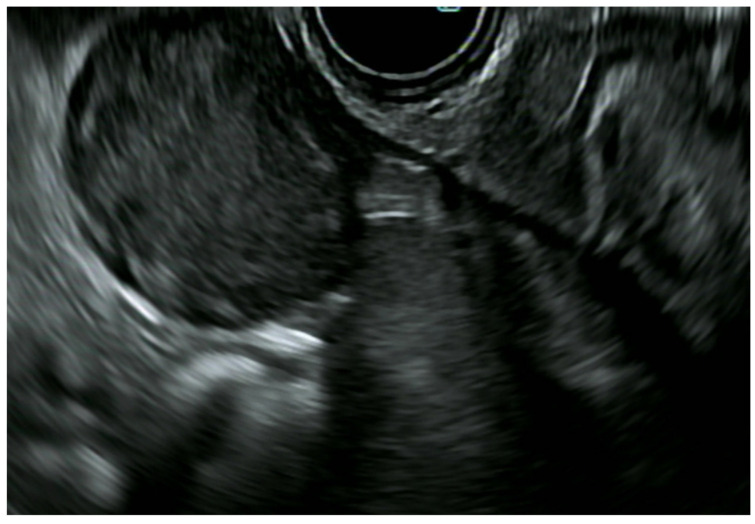
An exophytic GIST arising from the IV layer.

Mesenchymal tumors are the most frequent intramural lesions, comprising 54% of gastric SELs. The majority of them are GISTs and leiomyomas. GISTs account for 1/5 of GI tract sarcomas and represent 1–2% of all GI malignancies and more than a half of them are gastric (Figure 6) [104]. Other common SELs are lipomas, pancreatic rests, neuroendocrine tumors, schwannomas, varices, duplication cysts, and inflammatory fibroid lesions. Less common lesions include lymphoma, lymphangioma, glomus tumor, and submucosal metastases. Moreover, there are very rare mesenchymal lesions such as sarcomas, hemangiomas, inflammatory myofibroblastic tumors, plexiform myxofibromas, calcifying fibrous tumors, and desmoid fibromatosis. A rare entity is also the gastric wall abscess. We have to keep in mind that another potential gastric lesion mimicking an SEL is early gastric cancer, which sometimes can have an intact epithelium [105,106,107]. Thus, it is crucial to schedule a follow-up in SELs with inconclusive findings [108]. What we need to know for lesions’ differential diagnosis is: the wall layer of origin (by focusing on the transition zone between the normal gastric wall and the SEL), lesion echogenicity and homogeneity, vessels presence (by using Doppler), borders, size, CE-EUS behavior and elastography pattern, and signs of infiltration of adjacent organs and lymph nodes. Based on these features, the endosonographer can identify duplication cysts, varices, ectopic pancreas and lipomas. Other SELs’ diagnoses, such as GISTs, leiomyomas, schwannomas, granular cell tumor, and lymphoma, which typically can originate from the muscularis propria and are all hypoechoic on EUS, require histology, because some of them are malignant or harbor a malignant potential (Table 1). Differentiating between GISTs and leiomyomas is the most frequent challenge as these are the most seen II- and IV-layer lesions. A recent retrospective study aimed at finding factors significatively associated with GISTs rather than with leiomyomas included the following: EUS heterogeneity, non-cardia site, and older age were found to be significant independent factors [109]. The CT scan has a worse performance than EUS in a SEL setting.

A retrospective analysis in 53 SELs reveals that the overall accuracy of CT in histology is 50.9%: 74.2% in GISTs, 0% in leiomyoma, and 14.3% in ectopic pancreas, each of which is lower than that of EUS. The accuracy also decreases as the lesion diameter decreases [110]. According to the guidelines [46], EUS-guided sampling is indicated for hypoechoic SELs > 2 cm, as, below this limit, the risk of malignancy is very low. Furthermore, a histological examination is also indicated in the case of features suspicious for malignancy on EUS or endoscopy (ulceration, irregular borders, echogenic foci, cystic spaces, and adjacent lymph-node enlargement), regardless of their size. For small lesions (≤2 cm), a biopsy, resection, or surveillance is recommended, choosing on an individual basis. EUS-guided tissue acquisition should obviously be performed only if the result can change patient management [108]. EUS with FNA/FNB has a high accuracy, sensitivity, and specificity for lesions > 2 cm [111,112]. Moreover, EUS-FNB has a very high diagnostic accuracy in detecting GISTs (89–93.8%) vs. EUS-FNA (37–75%) and, in some cases, is notably also superior to FNA with ROSE [113,114,115].

Currently, we do not have any evidence which supports the use of a needle rather than another, so guidelines do not recommend a specific FNB needle type. We also have no evidence that an instrument is better than another to provide a better lesion visualization and histological specimen [116]. Additional biopsy methods such as mucosal-incision-assisted biopsy (MIAB) and single-incision needle-knife biopsy (SINK) have been developed. A recent randomized controlled trial with 47 patients and a retrospective study with 177 patients showed that MIAB had a greater diagnostic yield than EUS-FNA/FNB for SELs < 20 mm, but with a longer procedural time [117,118]. Thus, MIAB can be a reliable choice, especially in SELs < 20 mm [49,108], where a diagnosis through FNA/FNB is particularly challenging because SELs are generally hard and movable and, thus, are more difficult to puncture than pancreatic lesions. Another independent factor for nondiagnostic EUS FNA/FNB, other than lesion size, is the lesion location, with the gastric body as the “easier” location and the fundus and antrum as the worst ones, because the lesions are difficult to hold with a scope. In such cases, a cap-assisted forward-viewing endoscope is extremely helpful by fixing the lesion to puncture in the cap [119].

**Figure 7:**
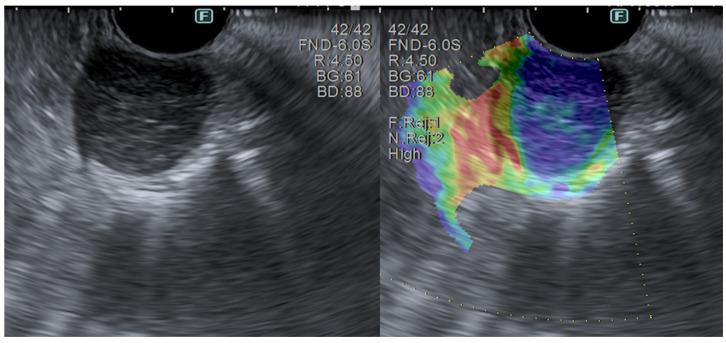
Elastography showing the high stiffness of a GIST.

Special SELs, in terms of diagnosis, are neuroendocrine tumors (NETs). Since NETs can arise from the muscularis mucosa, most times, forceps biopsies are diagnostic. When the lesion lies completely in the submucosal layer, then EUS-FNB is necessary [120]. Some studies revealed that CE-EUS is useful for distinguishing between GISTs and other SELs because GISTs are hypervascular lesions compared to other SELs. Hyperenhancement is strongly suggestive of GISTs with a 78–100% sensitivity, 60–100% specificity, and 60–100% accuracy, whereas hypoenhancement is associated to leiomyomas. CE-EUS can also help to predict GISTs’ malignant potential (by findings such as irregular vessels, a heterogeneous perfusion pattern, and the presence of non-enhancing spots). Moreover, GISTs are the stiffer SELs at elastography (Figure 7) [108]. Miniprobes can help with the visualization and study of small lesions (<2 cm), with the advantage of making a diagnosis at the same time of the upper GI endoscopy [5,121].

We also have promising outcomes from AI use in differentiating SELs. Minoda et al. demonstrated that AI has a better accuracy, sensitivity, and specificity in recognizing GIST from non-GIST SELs, independent of lesion size, in comparison to senior endoscopists, with an accuracy comparable to the results of FNB, thus assuming that we will replace FNB with AI [122]. Additionally, Kim et al. were able to differentiate between non-GIST lesions (leiomyomas from schwannomas) in 75% cases [123].

**Figure 8:**
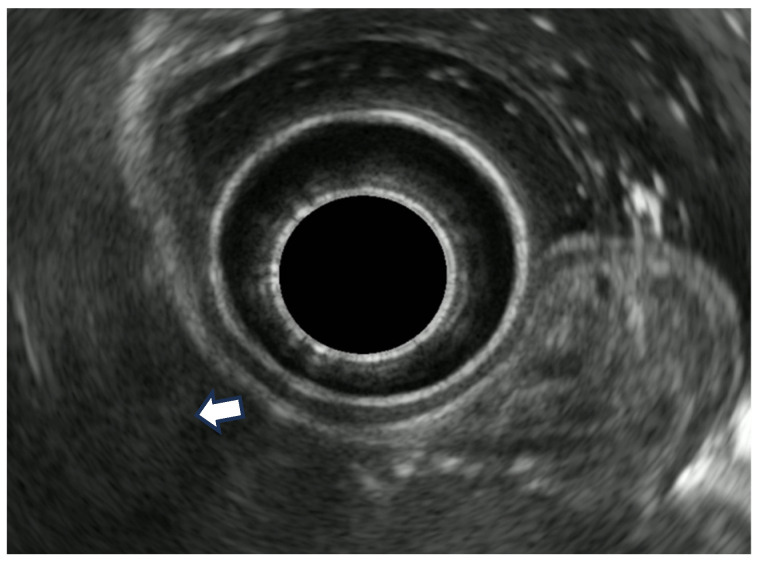
Ectopic pancreas in the III layer, with anechoic structures (arrow) without Doppler signal (pancreatic ducts).

Benign lesions such as leiomyoma, lipoma, schwannoma, and pancreatic rest (Figure 8) do not require follow-up. GIST management is more complicated as their malignant potential is determined by their size, mitotic activity, and the location (with a lower malignant potential for gastric ones). Since the mitotic activity is best defined after resection, the tumor size is a critical factor for their further management. For GISTs > 2 cm in size or with high-risk features, several guidelines recommend resection [46,49]. The European Society for Medical Oncology (ESMO) suggests, instead, resection regardless of size, because we have evidence that small GISTs (<2 cm) may also harbor an intermediate or high risk of progression [104,122,123,124]. An endoscopic resection may be suitable for the management of patients with small gastric undefined SELs (1–2 cm), with diagnostic and therapeutic purpose.

In cases of asymptomatic, hypoechoic SELs < 2 cm without high-risk features on EUS (lesions with a reported growth rate < 4% in 16–118 months), without a clear GIST suspicion, not tissue sampling but surveillance is mandatory. Most experts agree with an initial strict follow-up interval (e.g., EUS at 3–6 months) in order to assess lesion growth. Then, surveillance with EUS every 1–2 years for lesions between 1–2 cm and every 2–3 years for lesions < 1 cm is reasonable [49,125]. The best strategy (surveillance vs. treatment) in undefined lesions without a clear malignancy suspicion will be evaluated on a case-by-case basis, taking into account mainly the patient age, comorbidities, and preferences.

**Table 1:** Overview on gastric hypoechoic SELs. IHC: immunohistochemistry; SMA: smooth muscle actin (adjusted from [108]).

Type	Origin	EUS Features	Borders	Location	Malignant Potential	ICH
Glomus tumor	3rd/4th	Hypo/hyperechoic Hypervascular with internal echo	Sharp	Any	Rarely	SMA, vimentin, and laminin-positive. C-kit- and CD34-negative.
NET	1st/2nd/3rd	Hypo/hyperechoic	Sharp	Any	Yes	
GIST low risk	2nd/4th	Heterogeneous, hypervascular	Sharp, when benign	Any	Yes	C-kit- and DOG1-positive.
GIST high risk	2nd/4th	Heterogeneous with cystic space or echogenic foci	Irregular >3 cm	Any	Yes	C-kit- and DOG1-positive.
Leiomyoma	2nd/4th	Rarely multifocal	Sharp	Cardia	No	Desmin- and α-SMA-positive. C-kit- and CD34-negative.
Lymphoma	2nd/3rd/4th	Hypoechoic	Irregular	Any	Malignant	Dense lymphoid infiltrate.
Inflammatory fibroid polyp	2nd/3rd	Homogeneous, polypoid	Indistinct	Pylorus or distal antrum	No	Spindle and stellate stromal cells in a loose edematous stroma.
Ectopic pancreas	3rd/4th	Eterogeneous, with cysts or ducts inside	Indistinct	Antrum	Very rarely	Pancreatic acini, ducts, islets of Langerhans, and intervening connective tissue.
Schwannoma	4th	Homogeneous, sometimes with marginal halo	Sharp	Body	No	S100-positive. C-kit- and CD34-negative
Metastasis (breast, lung, kidney, ovary. and cutaneous melanoma)	Any	Hypoechoic	Irregular	Any	Malignant	Primitive tumor features

## 7. Endoscopic Rectal Ultrasound

Endoscopic rectal ultrasound (ERUS) allows the visualization of the anal canal and sphincters, the rectal wall and its adjacent structures, and the left iliac vessels, including enlarged lymph nodes when present. ERUS has, therefore, been applied for the evaluation of rectal mucosal lesions (rectal cancer staging and polypoid lesions assessment), subepithelial lesions, anal fistulas, and perianal abscesses, and, lately, to perform therapeutic interventions such as peri-rectal collections drainage [126,127,128]. It is generally performed using radial ultrasound scopes, which provide us with an easier exploration and identification of structures, but linear ultrasound scopes can also be used, allowing us to perform the FNA or FNB of enlarged nodes or other deep/extra-parietal lesions [129], and to guide therapeutic interventions [130]. Miniprobes can be used to perform an ultrasonographic evaluation of lesions throughout the entire colon and the distal ileum. Similarly, forward-viewing radial-array scopes have been developed to extend the field of ERUS evaluation to more proximal lesions, overcoming the limit of oblique-viewing echoendoscopes. However, the availability among endoscopy units and validation are still low for both these technologies [131].

## 8. Rectal Neoplastic Lesions

Colorectal cancer (CRC) is the third most commonly diagnosed malignancy and the second leading cause of cancer death worldwide [132]. Rectal cancer accounts for about 35% of CRCs in the European Union. Accurate staging is paramount in this setting, as it defines the most appropriate therapeutic path for the patient [133,134].

**Figure 9:**
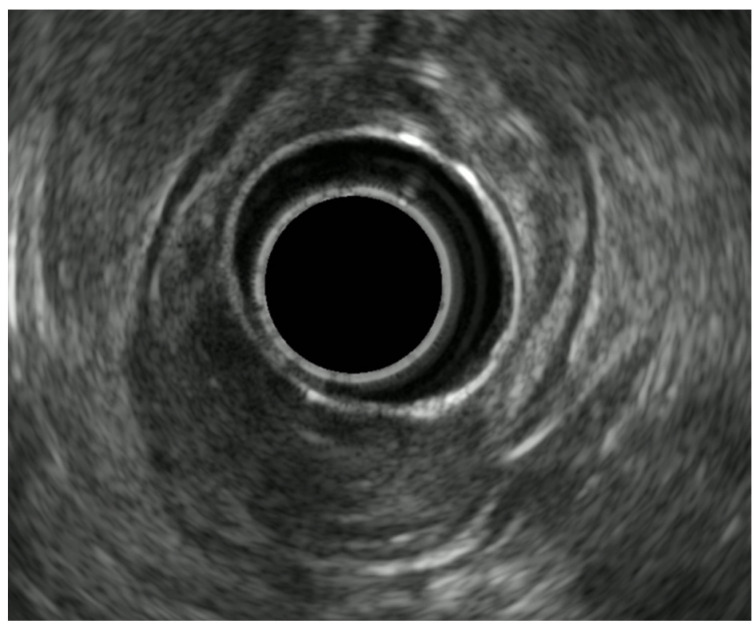
Advanced rectal cancer (uT3 with muscularis propria thickening and irregularity).

ERUS and pelvic magnetic resonance imaging (MRI) have traditionally been the main tools for rectal cancer loco-regional staging (T and N parameters). On ERUS, rectal neoplastic lesions appear as hypoechoic thickening originating from the mucosa (I–II layer), with a variable degree of disruption and involvement of the deeper layers depending on the T stage (Figure 9). Malignant lymph nodes are characterized by a combination of features such as a hypoechoic appearance, round shape, loss of the hyperechoic hilum, >5 mm short axis, and smooth borders. Data on the diagnostic accuracy of ERUS from the 2000s have been conflicting, as the accuracy for T staging ranged from 63% to 96% [135,136]. A 2009 systematic review concluded that there was a high accuracy of ERUS [137], but a large prospective German study again raised doubts on its diagnostic reliability, reporting an overall accuracy of 64.7%, with 18% cases of understaging and 17.3% cases of overstaging [138].

### 8.1. Rectal Superficial Cancer

Both the latest ESMO and National Comprehensive Cancer Network (NCCN) guidelines define MRI as the most accurate staging modality for rectal cancer [139]. ERUS plays a role mainly in superficial lesions. The higher spatial resolution of ERUS can indeed more reliably distinguish between T1 tumors, which can benefit from endoscopic/transrectal resection. According to the latest ESGE guidelines [40], after a rectal lesion endoscopic resection, if the single high-risk criterion is a submucosal invasion deeper than sm1 (well to moderately differentiated with no lymphovascular invasion and no grade 2 or 3 budding lesions), surveillance and/or chemo/radiotherapy can be preferred over surgery on an individual basis. It is well-known that ERUS cannot clearly distinguish a superficial from a deeper submucosal involvement; therefore, in this setting, EUS’ role is to exclude contraindications to an endoscopic approach, such as a T2 or N+ lesion. CE-EUS and elastography are useful tools also in this setting. A recent case series suggests that the use of CE-EUS might further increase the accuracy of ERUS in differentiating T1 from T2 tumors [140]. Elastography is also a promising add-on tool to enhance the differentiation of rectal adenomas from tumors, with a reported 84% accuracy [141].

### 8.2. Advanced Rectal Cancer

In this scenario (Figure 9), ERUS is only suggested when the pelvic MRI is contraindicated or inconclusive. Each imaging modality has its own strengths and weaknesses [139]. MRI has a higher accuracy in evaluating the neoplastic involvement of the mesorectal fascia, hence defining the circumferential resection margin (CRM) in more advanced lesions, which is extremely important both as a prognostic factor and in the surgical planning of rectal cancer treatment. In addition, ERUS is an operator-dependent procedure and it has been penalized by previous reports of a suboptimal lymph-node involvement assessment accuracy (70–75%) [142]. Nonetheless, accurate N staging for rectal cancer is a known issue, even after combining diagnostic modalities [143], and MRI can also overstage rectal cancer due to the desmoplastic reaction of adjacent structures [139]. A very recent systematic review and meta-analysis showed a comparable and high accuracy for T staging between ERUS (87%) and MRI (91%); similarly, the accuracy for N staging was very high with 93 and 92%, respectively [144].

As far as the restaging of rectal cancer after chemotherapy/radiation is concerned, the ERUS accuracy is hindered by post-therapy changes like edema, necrosis, or fibrosis, which frequently cause disease overstaging [136].

### 8.3. Rectal Subepithelial Lesions

SELs can be an incidental or symptomatic finding after colonoscopy or CT/MRI imaging. The ERUS accuracy in evaluating suspected SELs has been prospectively studied [145], yielding a high sensitivity (98%) in detecting intramural lesions, but a low specificity (64%) and an accuracy of only 48% compared to the FNB histology. The accuracy before FNB was lowest for III- and IV-layer hypoechoic lesions. The sensitivity for neuroendocrine tumors (NETs) is also high (94.4%), but the specificity is suboptimal (74.2%) with false-positive findings represented by inflammatory lesions, endometriosis, gastrointestinal stromal tumor (GIST), lymphoma, hemangioma, schwannoma, and metastasis [146]. Thus, histological confirmation is usually needed for SELs. EUS have been used to evaluate the residual neuroendocrine tissue in the case of the accidental removal of a neuroendocrine tumor deemed to be a hyperplastic polyp. In this setting EUS may be useful for excluding nodal disease, but scar removal by ESD seems to be the best option [147].

### 8.4. Inflammatory Bowel Disease (IBD) and Anal Incontinence

The main indication for ERUS in IBD patients is the evaluation of perianal Crohn’s disease, represented by fistulas and abscesses. The pelvic MRI is usually the first diagnostic examination, with exploration under anesthesia (EUA) being the gold standard for the assessment of fistulas. However, a 2012 meta-analysis [148] showed that the sensitivity of ERUS is comparable to that of MRI, while both methods suffered from a low specificity (43% and 69%, respectively). A more recent meta-analysis favored ERUS for inter-sphincteric and trans-sphincteric fistula identification and MRI for supra-elevator and extra-sphincteric fistulas [149]. ERUS, MRI, and EUA might be seen as complementary tools [150]. Fistulas are described according to Park’s classification and appear as a hypoechoic linear interruption of structures, often harboring hyperechoic spots, which represent gas bubbles and can be enhanced by the instillation of hydrogen peroxide [151]. The direct endoscopic visualization of a fistulous orifice in the mucosa and the possibility to monitor the healing of perianal disease represent additional advantages of ERUS over MRI [150].

ERUS can more reliably assess anal incontinence than digital anorectal examination, as it can evaluate the integrity of the internal (IAS) and external anal sphincter (EAS). ERUS has been compared to MRI with conflicting results, but its sensitivity for IAS injury was superior [152].

## Abbreviations

EUS: endoscopic ultrasound, FNA: fine-needle aspiration, FNB: fine-needle biopsy, CT: computed tomography.
